# Personal protective equipment used by obstetricians and obstetric nurses during the COVID-19 pandemic in Mansoura, Egypt

**DOI:** 10.12688/f1000research.110835.2

**Published:** 2022-07-04

**Authors:** Eman Khashaba, Abdel-Hady El-Gilany, Hend Shalaby, Rania El-Kurdy

**Affiliations:** 1 Assistant professor of Occupational Health and Industrial Medicine,Public health & Community medicine, Faculty of Medicine,Mansoura University, Mansoura, 35516, Egypt; 2 Professor of Public Health & Preventive Medicine,, Faculty of Medicine, Mansoura University, Mansoura, 35516, Egypt; 3 Professor of Obstetrics & Gynecology, Faculty of Medicine, Mansoura University, Mansoura, 35516, Egypt; 4 Lecturer of Woman’s Health & Midwifery Nursing, Faculty of Nursing,Mansoura University, Mansoura, 35516, Egypt

**Keywords:** PPE use- Obstetricians, Covid 19, Emergency labor, Intrapartum practice, occupational safety

## Abstract

**Background**: This study was done to describe the pattern of personal protective equipment (PPE) use, associated factors, and adverse events among obstetricians and obstetric nurses in obstetrics & gynecology departments.

**Methods**: A cross sectional study was conducted in Obstetrics & Gynecology departments in three hospitals (physician & nurses n=252) using an online Google form including demographic and occupational health data, type of available personal protective equipment during usual care, CS and emergency labor, infection control measures and hazards of full PPE use.

**Results **Full PPE use was 37.7% during CS and 34.9% during emergency labor. The significant predictors of wearing full PPE during CS were daily work hours > 8 hours and receiving formal training about PPE use. During CS & emergency labor most of HCws used sterile gloves and sterile fluid resistant gowns and surgical mask.to less extent used face shields or tight fitting googles and one tenth (11.8%) only used N95. The most common health effects of full PPE use was sense of heat (79.5%) **.**

**Conclusion: **During the COVID-19 pandemic more vigorous respiratory (N95 mask) and eye protection is required during aerosol-generating procedures. Formal training is an evident predictor for full PPE use.

## Introduction

COVID-19 is a global pandemic affecting all populations, and subsequent worldwide aggressive measures have been taken to mitigate the spread of the infection ( World Health Organization (WHO), 2020). According to a study on COVID-19 characteristics and predicting factors among healthcare providers in a developing country, approximately two-thirds of exposure occurred primarily during healthcare provision. Furthermore, one-fifth of the cases were confirmed COVID-19 cases; the majority of them had mild to moderate symptoms, with only 9.1 percent asymptomatic. Almost all became infected while on duty (97.4 percent). Infected patients (39%) were the most common source of infection, followed by colleagues (22.1%), household contacts (5.2%), and unknown sources (33.8 percent) ( El-Sokkary *et al.*, 2021).

Obstetricians have worked tirelessly for the past three decades to enhance women’s outcomes through the use of evidence-based medicine. The present COVID-19 epidemic has flooded this standardized method with a deluge of contradicting material, causing uncertainty in the birthing ward about recommended practices. As the infection spreads throughout the population, obstetricians are becoming more concerned ( Sichitiu & Desseauve, 2020).

Asymptomatic patients are contagious and thus are at a high risk of nosocomial infection ( Rothe *et al.,* 2020). Therefore, strict PPE usage for doctors and midwives is necessary at labor if universal screening is not performed ( Umazume *et al.*, 2020).

Regulating and adapting all aspects of infection prevention can be difficult in the unfortunate event of a maternal collapse. The delivery room is overloaded when many personnel simultaneously attempt to resuscitate the collapsed patient, perform a perimortem cesarean delivery, and resuscitate the newborn. The resuscitation team should don full PPE. The most common frequent & serious cross-infection to healthcare workers during outbreaks happened when first responders were not wearing the recommended PPE ( Schwartz and Graham, 2020).

Wearing N95 respirators can reduce clinical respiratory infections by 73 per 1,000 healthcare workers (HCWs). In laboratory-confirmed bacterial colonization, N95 respirators had a protective effect. N95 respirators were found to be more effective in preventing laboratory-confirmed respiratory viral infections and influenza-like illnesses. As regards protection of HCWs, no direct high-quality evidence was found on whether N95 respirators are better than surgical masks for from SARS-CoV-2 ( Iannone *et al.*, 2020).

Despite the protective PPE value, one study reported that physicians who work in the intensive care unit (ICU) and deal with such patients are naturally anxious. They would be unable to drink, eat, or go to the toilet for approximately 6 hours after wearing full PPE as required in the ICU. Taking off PPE after duty hours necessitates training and extreme caution to avoid self-infection ( Alsubaie *et al.*, 2019).

Worldwide, reports of PPE scarcity and unavailability are emerging. HCWs and the general press use social media to report on the reusing of PPE or the use of household and self-made items in place of PPE. There is limited evidence on the effectiveness of these practices, but they have occasionally been implemented on the advice of their employers or health organizations ( England, 2020; CDC, 2020). It is believed that no previous studies discuss the pattern of PPE utilization among obstetricians and obstetric nurses during the COVID-19 pandemic in our country.

### Study objectives

This study aims to describe the pattern of PPE use, associated factors, and adverse events among healthcare workers in obstetrics and gynecology departments.

## Methods

### Study design

Observational cross-sectional study with an analytic component.

### Study place and time

The study was conducted in Obstetrics & Gynecology Departments in three hospitals (one is affiliated to Mansoura University and two affiliated to the Ministry of Health & Population) from August 2020 to June 2021.

### Study participants

The study included on-duty obstetric physicians and nurses during the COVID-19 pandemic. Participation was voluntary and anonymous.

The sample size was calculated by openepi.com, an online sample size calculator. It was calculated according to the primary outcome of interest which is the anticipated frequency of full PPE use (30%) based on a pilot study of 100 subjects. The current study has a target population (~954 workers), power of the study (80%), and precision degree (5%). The sample size is 242, and 10% was added to cover nonresponse from online surveys. Thus, the final sample was 266 subjects.

### Data collection approach

The current study collected, first, personal communication between one of the authors and study subjects in selected hospitals to encourage participation and explain study objectives. Second, an online questionnaire was distributed ( *n* = 266) through posting Google links to WhatsApp personal accounts or WhatsApp groups of their departments. Snowball sampling technique (exponential non discriminative) was used by asking participants to share the link with other healthcare workers in the obstetrics and gynecology departments in their hospitals. Finally, the response rate was 252 (79.2%).

### Study tool

The questionnaire included demographic data; occupational history; type of available personal protective equipment during usual care, Cesarean section (CS), and emergency labor; infection control measures related to the use and discard of equipment; and hazards of full PPE during COVID-19 pandemic.

Operational case definition of full PPE use was adapted from the national baseline resources and recommendations of the International Society of Ultrasound in Obstetrics and Gynecology, Royal College of Obstetricians and Gynaecologists, Collège National des Gynécologues et Obstétriciens Français ( Poon *et al.,* 2020; Royal College of Obstetricians and Gynaecologists, 2021; Peyronnet *et al.,* 2020). The definition of PPE use in the current study included using gown with long sleeves, gloves (sterile gloves for CS and latex gloves for usual care), respiratory protection (N95 mask or FFP or surgical mask), eye protection (face shield or goggle), and foot protection (overshoes or safety boots).

### Ethical considerations

The study was approved by the Research Ethics Committee of the Faculty of Medicine of Mansoura University (code number: R/20.7.925). Informed verbal consent was obtained from the administration of hospitals affiliated with the Ministry of Health and Population. All study participants were assured of the confidentiality and anonymity of the data at the start of Google form, and participation was voluntary.

### Statistical analysis

Data were analyzed using SPSS, version 23. Categorical variables are presented as numbers and percentages, and the chi-squared test was used for comparison between groups. Crude odds ratios and their 95% confidence intervals (CIs) were calculated. Moreover, quantitative variables are presented as means and standard deviations. Binary stepwise logistic regression analysis was used to determine the independent predictors of full PPE use as the dichotomous outcome variable. Variable found to have statistical significance in bivariate analysis were entered into the logistic regression analysis using a Forward-Wald model. Model fitness by Hosmer and Lemeshow test was non-significant ( *p* value > 0.05) which indicates Good fit for model. Adjusted odds ratios and their 95% CIs were calculated. A *p* value ≤ 0.05 was statistically significant.

## Results

### Demographic characteristics

The study included 252 healthcare workers in the obstetrics and gynecology departments. Most of the studied participants were females (76.2%), has a mean age of 32.2 (9.9), and nearly half of them are from urban areas (47.2%). About two-thirds (57%) of the studied HCWs were from Mansoura University Hospitals. More than half were nurses (56.7%) and 37.7% were physicians. The median work duration in years and median daily work hours were 7 years and 8 h, respectively. One-fifth of them (20.2%) reported work in hospital isolations. Most of them reported exposure to COVID-19 patients (75.5%). However, the polymerase chain reaction-confirmed COVID-19 infection was about one-third (27.3%) of the studied HCWs. This percentage is exactly half of those were symptomatic workers (50.9%; data are not tabulated).

### Factors associated with full PPE use

Full PPE use was 37.7% and 34.9% during CS and emergency labor, respectively. Full PPE use during CS was significantly associated with longer work hours (≥8 h), working in hospital isolation, and receiving formal training about PPE use ( *p* < 0.05). Moreover, it was associated with working in hospital isolations and receiving formal training during emergency labor ( *p* < 0.05; Table 1) ( Khashaba, 2022).

**Table 1.** Factors associated with full PPE use among study participants during CS & emergency labor.

Associated factors	Total	Full PPE during CS		Full PPE during emergency labor	
		n (%)	COR (95%CI)	n (%)	COR (95%CI)
**Overall**	**252**	95 (37.7%)	----	88 (34.9%)	**---**
**Age**					
≤30 (r)	117	48 (41.)	0.7 (0.4-1.2)	43 (36.8)	0.8 (0.5-1.4)
>30	135	47 (34.8)		45 (33.3)	
**Sex**			0.8 (0.4-1.4)		
Male (r)	60	25 (41.7)		25 (41.7)	0.6 (0.3-1.2)
female	192	70 (36.5)		63 (32.8)	
**Educational level**					
Technical institute (r)	135	47 (34.8)		43 (31.9)	
Bachelor	47	17 (36.2)	1.06 (0.5-2.1)	19 (40.4)	1.4 (0.7-2.8)
Post graduate studies	70	31 (44.3)	1.4 (0.8-2.6)	26 (37.1)	1.2 (0.6-2.3)
**Type of hospital** ^#^					
University (r)	139	58 (41.7)		48 (34.5)	
MOH	105	36 (34.3)	0.7 (0.4-1.2)	36 (34.3)	0.9 (0.6-1.7)
**Job description**					
Physician (r)	95	41 (43.2)		36 (37.9)	
Nurse	157	54 (34.4)	0.6 (0.4-1.16)	52 (33.1)	0.8 (0.4-1.4)
**Duration of work**					
≤7 years (r)	146	54 (37)		55 (37.7)	
>7 years	106	41 (38.7)	1.07 (0.6-1.8)	33 (31.1)	0.7 (0.4-1.2)
**Daily work hours**					
< 8 hours (r)	114	31 (27.2)		36 (31.6)	
≥ 8 hours	138	64 (46.4)	2.3 (1.3-3.9) *	52 (37.7)	1.3 (0.7-2.2)
**Work in isolation hospital**					
No (r)	201	68 (33.8) *		64 (31.8)	
Yes	51	27 (52.9)	2.2 (1.2-4.1) *	24 (47.1)	1.9 (1.0-3.5) *
**Exposed to covid 19 infection**					
No (r)	64	24 (37.5)		22 (34.4)	
Yes	188	71 (37.8)	1.01 (0.5-1.8)	66 (35.1)	1.0 (0.5-1.8)
**Confirmed covid 19 infection**					
No (r)	194	70 (36.1)		64 (31.8)	
Yes	58	25 (43.1)	1.3 (0.7-2.4)	24 (47.1)	1.4 (0.7-2.6)
**Received formal training**					
No (r)	92	25 ( 27.2)	2.08 (1.2-3.6)	22 (23.9)	2.2 (1.2-3.9)
Yes	160	70 (43.8)		66 (41.3)	

^#^ Missing data from type of hospital 11 subjects, row percent is considered, COR: crude odds ratio.

Multivariable logistic regression model for predictors of wearing full PPE during CS revealed that >8 h daily work hours (odds ratio [OR], 2.3; 95% CI, 1.3–3.8) and receiving formal training about PPE use (OR, 2.03; 95% CI, 1.16–3.5) were statistically significant among obstetricians during the COVID-19 pandemic. A significant predictor of full PPE use during emergency labor was receiving formal training only ( *p* < 0.05; OR, 2.2; 95% CI, 1.2–3.9. The regression model predicted 64.3% & 65.1% of variation in full PPE use during CS and emergency labor (dependent variable); Table 2).

**Table 2.** Logistic regression analysis for predictors of full PPE use during CS.

Predictors	Full PPE during CS			Full PPE during emergency labor		
	β	p value	Adjusted OR (95%CI)	β	p value	Adjusted OR (95%CI)
**Daily work hours**						
< 8 hours (r)			1	-	-	-
≥ 8 hours	0.81	0.003	2.3 (1.3-3.8)			
**Received Formal training**						
No (r)			1			1
Yes	0.71	0.01	2.03 (1.16-3.5)	0.8	0.006	2.2 (1.2-3.9)
**Constant**	-1.45			-1.15		
**Percent correctly predicted**	64.3			65.1		
**Model χ ^2^**	16.1; 0.001			7.9; 0.005		

### Pattern of PPE use & infection control measures among study participants

During CS, most healthcare workers used sterile gloves (88%), sterile fluid-resistant gowns (75.6%), and surgical masks (79.2%). About half (52.6%) used face shields. About one-third of them used tight-fitting goggles (34.3), and one-tenth (11.8%) only used N95. Moreover, during emergency labor, most healthcare workers used sterile gloves (81.1), sterile fluid-resistant gowns (70.7), and surgical masks (83.7%). Washing gloves and reusing gowns were reported in 26.8% and 14.8% of HCWs, respectively. Most of the study subjects reported changing gloves between patients (87.7%). More than two-thirds of the subjects followed the correct sequence of donning, removing PPE, and receiving formal training on the use of PPE (67.1%, 66.4%, and 63.5%, respectively; Table 3).

**Table 3.** Pattern of PPE use & infection control measures among study participants.

Variable (valid responses)	Study subjects n (%)
**PPE during CS**	
Wear sterile fluid resistant gown (n = 248)	189 (75.6)
Wear surgical mask (n = 241)	198 (79.2)
Wear N95 (n = 241)	29 (11.8)
Wear FFP3 (n = 240)	8 (3.3)
Wear face shield (n = 245)	132 (52.6)
Wear tight fitting goggles (n = 247)	86 (34.3)
Wear foot protective equipment (n = 248)	162 (64.8)
Wear sterile gloves (n = 248)	220 (88)
**PPE during emergency labor**	
Wear sterile fluid resistant gown (n = 248)	176 (70.7)
Wear surgical mask (n = 246)	206 (83.7)
Wear N95 (n = 246)	21 (8.5)
Wear FFP3 (n = 246)	10 (4.1)
Wear mask before entering room (n = 234)	210 (89.4)
Wear tight fitting goggles or face shield (n = 248)	79 (31.7)
Wear foot protective equipment (n = 242)	212 (87.6)
Wear sterile gloves (n = 248)	202 (81.1)
Remove mask before leaving (n = 248)	170 (68.0)
Limited number of staff per case (n = 247)	205 (83.0)
**General infection control measures**	
Changed gloves after use in between patient (n = 251)	221 (87.7)
Wash gloves when visibly contaminated (n = 251)	67 (26.8)
**Reused gowns (n = 183)**	27 (14.8)
Avoid touching your face with contaminated gloves (n = 248)	226 (91.1)
Avoid touching environmental surfaces with contaminated gloves (n = 251)	222 (88.4)
Hand hygiene immediately after patient touching or leaving examination room (n = 248)	218 (87.9)
Discard gloves in the nearest appropriate receptacle "red bags" (n = 249)	220 (88.4)
**Follow correct sequence of donning PPE (n = 246)**	165 (67.1)
**Follow correct sequence of removing PPE (n = 250)**	166 (66.4)
**Received formal training on use of PPE (n = 252)**	160 (63.5)

Percent is calculated according to total valid responses (between parenthesis).

### Adverse health effects of full PPE use

**Figure 1.**
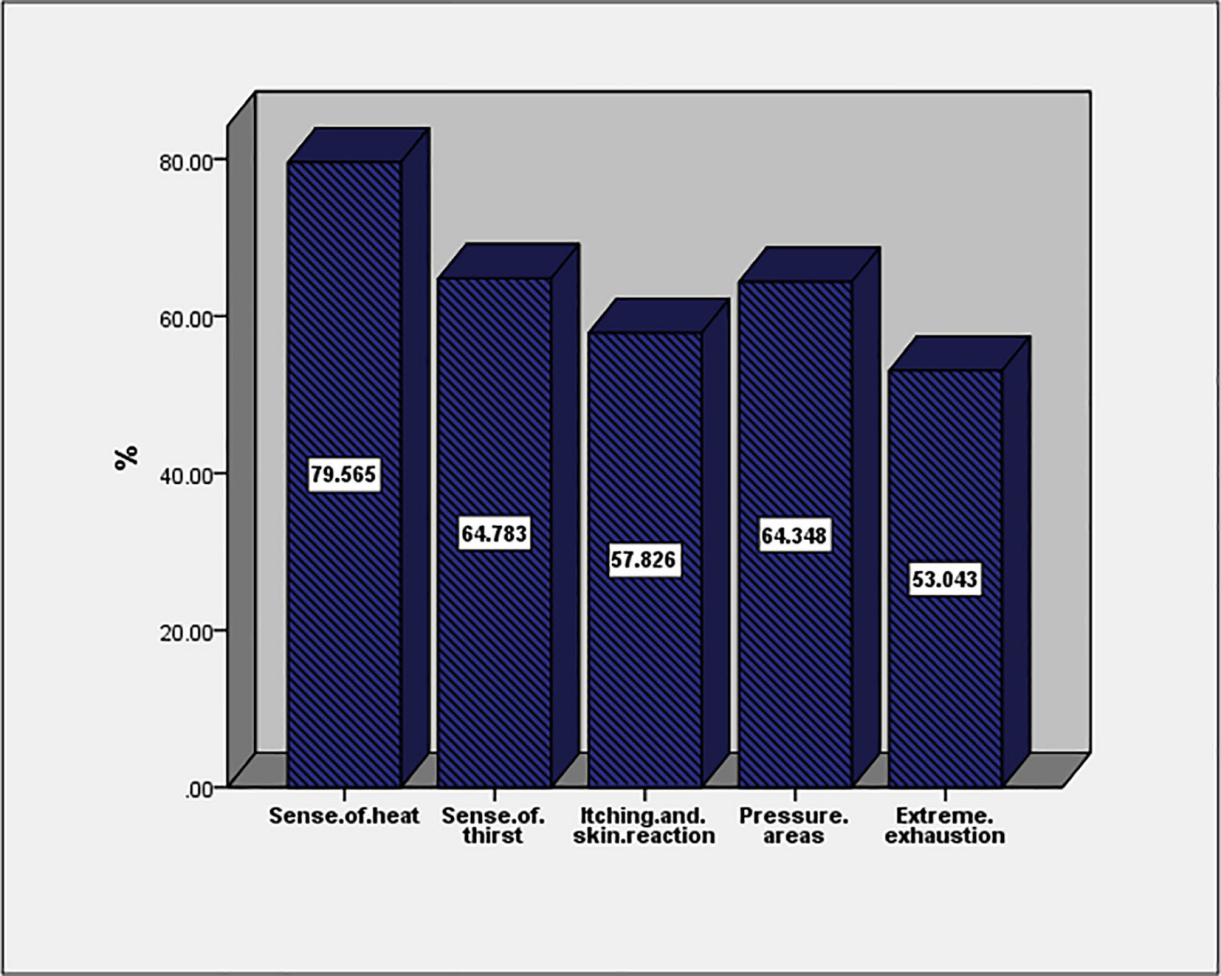
Adverse effects of full PPE use among study participants.

The most common health effect of full PPE use is a sense of health (79.5%) followed by a sense of thirst and pressure areas (64.7% and 64.3%, respectively; Figure 1). More than two-thirds of affected HCWs (68.3%) had more than two symptoms. Adverse events were not associated with age, gender, job description, duration of work in years, work hours, or work in hospital isolations ( *p* > 0.05; data are not tabulated).

## Discussion

This is the first study on PPE use among healthcare workers in the obstetrics and gynecology departments during the COVID-19 pandemic in Egypt. PPE use is influenced by the workload of HCWs as a matter of hours and patients, baseline resources in these hospitals, and stockpiling of personal equipment and safety training for those HCWs ( Tabah *et al.*, 2020).

The present study showed that full PPE use was 37.7% and 34.9% during CS and emergency deliveries, respectively. Full PPE use during CS was significantly associated with longer work hours (≥8 h) and formal training on PPE use. A different description for the pattern of full PPE per facility was noticed by a nationwide survey in Japan including core facilities and affiliated hospitals of obstetrics and gynecology training programs. Authors reported that full PPE was used by doctors and midwives in 7.1% and 6.8% of facilities, respectively, taking into consideration the different definitions of full PPE use in both studies.

The present study showed that most healthcare workers used sterile gloves, sterile fluid-resistant gowns, and surgical masks during CS and emergency labor. Fewer HCWs used face shields and goggles. Only one-tenth used N95 due to the absence of baseline resources of these hospitals. Of the HCWs, less than one-third reported washing gloves (26.8%) and reusing gowns (14.8%). Surgical masks and N95 respirators are the most consistent and complete support measures used by healthcare personnel, according to a Cochrane-approved systematic review on physical interventions to reduce respiratory virus transmission undertaken in 2011. N95 respirators are non-inferior, according to the highest quality cluster-randomized controlled studies included in this systematic review ( Jefferson *et al.*, 2011).

Another web-based survey conducted by Tabah *et al.* (2020) and distributed worldwide in April 2020 among ICU HCWs found that more than half of the respondents (52%) reported that at least one piece of the standard PPE is not available, and 30% reported that at least a piece of single-use PPE was being reused or washed as a result of shortages. Most of the available PPEs were designed for single-use and brief duration. Hence, the authors reported that urgent design and manufacture of PPE that can be safely worn and remains effective for extended durations are needed.

Similarly, the first nationwide Japanese survey reported that N95 masks and goggles or face shields were out of stock in 6.5% and 2.7% of facilities, respectively. In addition, disposable N95 masks and goggles or face shields were reused after resterilization in 12% and 14% of facilities, respectively ( Umazume *et al.*, 2020).

Masks and respirators play a role in the protection of health workers according to high level of agreement among key agencies. However, there are current differences between these agencies in terms how and when the different products are used. Different recommendations have been made by the World Health Organization (WHO), the US Centers for Disease Control and Prevention (CDC), and other leading health organizations. For example, the WHO recommends using N95, FFP2, FFP3 standards or equivalent in care settings for COVID-19 patients where aerosol-generating procedures are used and medical masks are used in the absence of aerosol-generating procedures ( WHO, 2020). In contrast, the US Centers for Disease Control and Prevention ( Center for Disease Control and Prevention, 2020) recommend using respirators during both routine care of COVID-19 patients and high-risk situations.

In this study, more than two-thirds of the subjects followed the correct sequence of donning and removing PPE and receiving formal training on PPE use (67.1%, 66.4%, and 63.5%, respectively). These results came lower than those observed among ICU workers in a worldwide web-based survey who found that most of the respondents (83%) had formal training in PPE use. This included training at the start of the institution (13%) and within the last two months (60%) due to the COVID-19 pandemic ( Tabah *et al.*, 2020).

In addition, current study results revealed that receiving formal training on PPE use among obstetric physicians and nurses was a significant predictor of full PPE use together during CS and emergency labor. These results are in agreement with an Italian study on a cross-section of physicians which reported that access to adequate information on the use of PPE was associated with a better ability to perform donning and doffing procedures as an example for proper PPE use ( Savoia *et al*., 2020).

The most common health effects of full PPE use in the current study is a sense of heat (79.5%) followed by a sense of thirst and pressure areas (64.7 and 64.3%, respectively). More than two-thirds of affected HCWs (68.3%) had more than two symptoms. These results were different from the results found in ICU workers during the pandemic where 80% had adverse events including heat (51%), thirst (47%), pressure areas (44%), headaches (28%), inability to use the bathroom (27%), and extreme exhaustion (,20%). They were all associated with longer duration of shifts wearing PPE. However, in the current study, adverse events were not associated with age, gender, job description, duration of work in years, work hours, or work in hospital isolation ( Tabah *et al.*, 2020).

## Conclusions

Most obstetricians and obstetric nurses used surgical masks, gloves, gowns, foot protection, and, to a lesser extent, N95, goggles, and face shields. Work hours (>8 h) and formal training were significant predictors for full PPE use.

Based on the findings of the current study, adding N95 or FFP mask and eye protection for safety programs in obstetrics and gynecology departments during aerosol-generating procedures is recommended. Also, reducing long shift work hours that may lead to adverse events due to full PPE use is recommended. Providing simple training videos about different types, proper PPE donning and removal, disinfection of reused equipment, and hand hygiene frequently as part of CME hours of physicians and nurses are required to cope with this emerging threat.

### Limitations of the study

The evaluation of PPE sufficiency during the COVID-19 pandemic was not assessed in this study. Further research is required to address PPE protective value in infected HCWs compared to non-infected controls.

## Data availability

### Underlying data

Harvard Dataverse: Personal protective equipment use by obstetricians and obstetric nurses during the COVID-19 pandemic in Mansoura, Egypt, https://doi.org/10.7910/DVN/XQRZB4 ( Khashaba, 2022).

This project contains the following underlying data:

- D.Eman ppe_obestritians.tab

### Extended data

Harvard Dataverse: Personal protective equipment use by obstetricians and obstetric nurses during the COVID-19 pandemic in Mansoura, Egypt, https://doi.org/10.7910/DVN/XQRZB4 ( Khashaba, 2022).

This project contains the following extended data:

- English Questionnaire PPE.pdf

Data are available under the terms of the Creative Commons Zero “No rights reserved” data waiver (CC0 1.0 Public domain dedication).
