# Supplementary material for: Personal protective equipment used by obstetricians and obstetric nurses during the COVID-19 pandemic in Mansoura, Egypt
Source: F1000Res. 2022 Jul 4;11:413. Originally published 2022 Apr 12. [Version 2] doi: 10.12688/f1000research.110835.2 (PMC9274016; doi:10.12688/f1000research.110835.2)
Supplement: Peer review history

# Peer review: Reviewer response for version 2

**DOI:** 10.5256/f1000research.135686.r143061

**Published:** 2022-07-11

**Authors:** Indranil Saha

**Affiliations:** 1 ICMR-Centre for Ageing and Mental Health (I-CAM), Indian Council of Medical Research, Kolkata, West Bengal, India

The authors have satisfactorily answered and responded to all my previous queries. Now the revised manuscript can be indexed.

Is the work clearly and accurately presented and does it cite the current literature?

Yes

If applicable, is the statistical analysis and its interpretation appropriate?

Partly

Are all the source data underlying the results available to ensure full reproducibility?

Yes

Is the study design appropriate and is the work technically sound?

Yes

Are the conclusions drawn adequately supported by the results?

Yes

Are sufficient details of methods and analysis provided to allow replication by others?

Partly

Reviewer Expertise:

Non-communicable diseases, Mental Health, ageing, communicable diseases

I confirm that I have read this submission and believe that I have an appropriate level of expertise to confirm that it is of an acceptable scientific standard.

# Peer review: Reviewer response for version 1

**DOI:** 10.5256/f1000research.122487.r136541

**Published:** 2022-06-28

**Authors:** Ibrahim Kabbash

**Affiliations:** 1 Department of Public Health and Community Medicine, Faculty of Medicine, University of Tanta, Tanta, Egypt

The articles discusses the issue of protective equipment use among health care workers. The lessons learnt from the field can be implemented to control the infection. Here I would like to raise some queries, which need to be clarified from the authors:

1. The sampling method technique was referred to as snowballing by the authors; however, it is actually a convenient sample as it does not meet the criteria for snowballing.
2. How did the authors identify the links of social media they used for recruiting participants to ensure representativeness of their sample to the target population?
3. How did the authors motivate the participants to share the data collection, and what was the duration to reach the desired sample size?

Overall the manuscript is well written. The authors have responded satisfactorily to the comments provided the other reviewer.

Is the work clearly and accurately presented and does it cite the current literature?

Yes

If applicable, is the statistical analysis and its interpretation appropriate?

Yes

Are all the source data underlying the results available to ensure full reproducibility?

Yes

Is the study design appropriate and is the work technically sound?

Yes

Are the conclusions drawn adequately supported by the results?

Yes

Are sufficient details of methods and analysis provided to allow replication by others?

Yes

Reviewer Expertise:

epidemiology of infectious diseases, HIV/AIDS, reproductive health

I confirm that I have read this submission and believe that I have an appropriate level of expertise to confirm that it is of an acceptable scientific standard.

# Response

**Published:** 2022-06-29

**Authors:** Eman Khashaba

**Affiliations:** Mansoura University, Egypt

**Response to reviewer** **’** **s comments**

**Thanks a lot for your valuable and accurate comments **

1. The sampling method technique was referred to as snowballing by the authors; however, it is actually a convenient sample as it does not meet the criteria for snowballing.
**Reply: Thanks for your comment. Actually, snowball sampling technique was used in this study. The questionnaire was distributed as on line Google form through what app groups of target population. Direct interview or self-reported questionnaires were not feasible due to lockdown of COVID-19 and strict hospitals policies of minimizing personal contacts with health care workers. The first subject was recruited to the sample then he provides multiple referrals for research.**
** Further clarification of sampling methods was added based on the comments of the first reviewer.**
2. How did the authors identify the links of social media they used for recruiting participants to ensure representativeness of their sample to the target population?
**Reply: Through** **WhatsApp** **groups of related departments whenever available or from one to his colleagues in the same department.**
3. How did the authors motivate the participants to share the data collection, and what was the duration to reach the desired sample size?
**Reply: In general, participation was voluntary and the response rate was 252 (79.2%).** **The personal protection is crucial part in occupational health & safety of HCws during such pandemic. This research stressed on the value of personal protection among obstetricians & nurses. **
**Duration already present in the manuscript from August 2020 to June 2021**
** About 1 year.**

# Peer review: Reviewer response for version 1

**DOI:** 10.5256/f1000research.122487.r134790

**Published:** 2022-04-28

**Authors:** Indranil Saha

**Affiliations:** 1 ICMR-Centre for Ageing and Mental Health (I-CAM), Indian Council of Medical Research, Kolkata, West Bengal, India

The article is pertinent in the level of policy implications. The lessons learnt from the field can be implemented to control the infection. Here I have raised some queries, which need to be clarified from the authors.

 The methodology section needs to be elaborated further. Since three hospitals were studied, what was the representation of the study population in the final sample itself? Moreover, were the original proportion of doctors and nurses in these 3 hospitals maintained in the final sample?

 Ideally, two sampling frames are ideal, or one sampling frame containing both doctors/nurses.

 Table 1: Subheading is absent. Please insert. 

 Why was the snowball sampling technique used in this study? It is generally used to reach hard to reach study subjects. That is not the case in this study. 

 Moreover, which type of snowball sampling technique was used - Linear/Exponential, Non-discriminative/Exponential discriminative? 

 The output of the logistic regression was also incomplete. What about the model fitness? The variation of dependent variables explained from independent variables? What proportion can be explained from the model? Please mention as running text in the results section. 

 The final model was a multivariable logistic binary regression model. Please mention this. 

 I would recommend replacing the term 'study subjects' with 'study participants'.

Is the work clearly and accurately presented and does it cite the current literature?

Yes

If applicable, is the statistical analysis and its interpretation appropriate?

Partly

Are all the source data underlying the results available to ensure full reproducibility?

Yes

Is the study design appropriate and is the work technically sound?

Yes

Are the conclusions drawn adequately supported by the results?

Yes

Are sufficient details of methods and analysis provided to allow replication by others?

Partly

Reviewer Expertise:

Non-communicable diseases, Mental Health, ageing, communicable diseases

I confirm that I have read this submission and believe that I have an appropriate level of expertise to confirm that it is of an acceptable scientific standard, however I have significant reservations, as outlined above.

# Response

**Published:** 2022-04-30

**Authors:** Eman Khashaba

**Affiliations:** Mansoura University, Egypt

**Response to reviewer** **’** **s comments**

**Thanks a lot for the valuable and informative comments.**

 The methodology section needs to be elaborated further. Since three hospitals were studied, what was the representation of the study population in the final sample itself? Moreover, were the original proportion of doctors and nurses in these 3 hospitals maintained in the final sample?

**Reply: The actual number of doctors and nurses in each hospital is not of concern as we aimed to complete the sample size calculated. Also the number on duty is not fixed due to frequent sick leaves, and many of them are not regular users of mass media to complete questionnaire in due time. In general, the number of the nurses is more than the doctors in original sample.**

 The proportion of each hospital was not kept in the final sample.

 This can be added TO STUDY LIMITATIONS.

 Ideally, two sampling frames are ideal, or one sampling frame containing both doctors/nurses.

**Reply: For our study, it is one sampling frame containing both doctors/nurses**

 Table 1: Subheading is absent. Please insert.

**Reply:** **Yes I agree we can add "associated factors".**

 Why was the snowball sampling technique used in this study? It is generally used to reach hard to reach study subjects. That is not the case in this study.

**Reply**: **The questionnaire was distributed on line with a Google form through WhatsApp groups of the target population. Direct interview or self-reported questionnaires were not feasible due to being in a lockdown as a result of the COVID-19 pandemic, and strict hospitals policies of minimizing personal contacts with health care workers.**

 Moreover, which type of snowball sampling technique was used - Linear/Exponential, Non-discriminative/Exponential discriminative? 

**Reply:** **The type of snowball sampling technique is Exponential non discriminative (I agree to add this type in the published manuscript).** **The first subject recruited to the sample provides multiple referrals** **for participation in research.**

 The output of the logistic regression was also incomplete. What about the model fitness? The variation of dependent variables explained from independent variables? What proportion can be explained from the model? Please mention as running text in the results section. The final model was a multivariable logistic binary regression model. Please mention this.

**Reply:**

1. **Model fitness by Hosmer and Lemeshow test was non-significant ( p value>0.05) which indicates Good fit for model. Nagelkerke R ^2^ was less than 1 for both models.**
2. **For the comment on multivariable logistic regression, I agree to modify this comment in results section.**

**The necessary changes will be as follows:**

 Multivariable logistic regression model for predictors of wearing full PPE during CS revealed that >8 h daily work hours (odds ratio [OR], 2.3; 95% CI, 1.3–3.8) and receiving formal training about PPE use (OR, 2.03; 95% CI, 1.16–3.5) were statistically significant among obstetricians during the COVID-19 pandemic. A significant predictor of full PPE use during emergency labor was receiving formal training only ( *p* < 0.05; OR, 2.2; 95% CI, 1.2–3.9;). The regression model predicted 64.3% & 65.1% of variation in full PPE use during CS and emergency labor (dependent variable).

 I would recommend replacing the term 'study subjects' with 'study participants'.

**Reply:** **I agree with you it can be replaced in the published manuscript &table headings).**
